# Turkey National Mesothelioma Surveillance and Environmental Asbestos Exposure Control Program

**DOI:** 10.3390/ijerph14111293

**Published:** 2017-10-25

**Authors:** Selma Metintaş, Hasan Fevzi Batırel, Hasan Bayram, Ülkü Yılmaz, Mehmet Karadağ, Güntülü Ak, Muzaffer Metintaş

**Affiliations:** 1 Medical Faculty Department of Public Health, Eskisehir Osmangazi University, Eskisehir 26040, Turkey; selmametintas@hotmail.com; 2 Lung and Pleural Cancers Research and Clinical Center, Eskisehir Osmangazi University, Eskisehir 26040, Turkey; guntuluak@gmail.com; 3 Medical Faculty Department of Chest Surgery, Marmara University, İstanbul 34722, Turkey; hfevzi@hotmail.com; 4 Medical Faculty Department of Chest Diseases, Koç University, İstanbul 34450, Turkey; hasantoraks@gmail.com; 5 Medical Faculty Department of Chest Diseases, Health Science University, Ankara 06430, Turkey; ulkuyilmazdr@gmail.com; 6 Medical Faculty Department of Chest Diseases, Uludağ University, Bursa 16059, Turkey; mehmetkarada@gmail.com

**Keywords:** mesothelioma, environmental asbestos exposure, epidemiology, asbestos

## Abstract

Malignant mesothelioma (MM) is an important health problem due to ongoing asbestos exposure. Environmental asbestos exposure leads to a high risk of MM in Turkey. The Turkish Mesothelioma Working Group and the Turkish Public Health Institute designed and performed the Turkey National Mesothelioma Surveillance and Environmental Asbestos Exposure Control Program (TUNMES-EAECP). The aim of this study was to analyze the results of the TUNMES-EAECP. Patients diagnosed with MM (code C45.0–C45.9) between 2008 and 2012 were identified. The “from case to the field” method was used to determine the villages with current or previous asbestos exposure. Special public health teams took soil samples from these villages, which were then examined using an X-ray diffractometer. Direct Standardized Average Annual Mesothelioma Incidence Rate (AMIR) and relative risk (RR) of MM were calculated. Finally, a projection on the incidence of MM between 2013 and 2033 was made. The number of confirmed MM cases was 5617 with a male to female ratio of 1.36. Mean age was 61.7 ± 13.4 (20–96) years. The median survival was eight (95% CI 7.6–8.4) months. Asbestos exposure continues in 379 villages, with 158,068 people still living in high risk areas. The standardized AMIR was 2.33/100,000 per year. The risk of MM was higher in males, in both sexes over the age of 40, in asbestos-containing provinces, and in those where the TUNMES was organized. Among the population with continuing asbestos exposure in rural areas, the number of MM cases between 2013 and 2033 was estimated as 2511. As such, the incidence of MM in Turkey is as high as in industrialized countries. Asbestos exposure in rural areas continues to be a serious problem in Turkey, which obviates the necessity for effective preventive measures.

## 1. Introduction

Malignant mesothelioma (MM) is a rare tumor, which can affect the pleura, peritoneum, and pericardium [1]. The majority of cases are linked to asbestos exposure, some of which in Turkey are due to erionite exposure [2,3,4,5]. It is a continuing health problem for communities that have occupational or environmental asbestos, or asbestos-like carcinogenic fiber exposure [6,7,8].

It has been well demonstrated that environmental exposure to asbestos through the use of asbestos-contaminated soil mixtures causes a high risk of MM in the rural regions of Turkey. The villagers in these mainly agricultural regions use this soil as a whitewash or plaster material (white stucco) for walls, as insulation and water proofing, floors and roofs, for baby powder, and also in pottery [3,4,9]. Environmental asbestos exposure is still present, although it tends to decline along with socioeconomic developments.

MM due to erionite exposure has been seen among people living in three villages around Cappadocia in Turkey [10]. Erionite, a fibrous silicate like asbestos, has been detected within some stones used for building houses in these villages where erionite was reported to be highly carcinogenic with an increased risk of MM [10,11].

The establishment of registries on cancers has been quite recent in Turkey. The Institute for Health Metrics and Evaluation; The Global Burden of Disease projection for the MM incidence in Turkey was reported as 1.06 per 100,000 people among males, and 0.39 per 100,000 people among females [12]. On the other hand, local findings reported from the rural areas—where asbestos or erionite exposure was highly prevalent—modestly indicate an underestimation of the impact of the problem. In a cohort composed of villagers who have certainly been exposed to environmental asbestos, the average annual mesothelioma incidence rate was determined as 114.8/100,000 person/year for men, and 159.8/100,000 person/year for women [3]. The same rates were determined as 639/100,000 person/year for men, and 1.267/100,000 person/year for women from another cohort, including villagers who were exposed to environmental erionite [11]. However, it was not possible to make a comparison or generalize the data among regional studies in Turkey due to the diversity of the methods used.

These limitations led to the authors of this paper to establish the Turkish Mesothelioma Working Group (TMWG) to conduct nationwide studies. TMWG and the Turkish Public Health Institute prepared and performed Turkey’s National Mesothelioma Surveillance and Environmental Asbestos Exposure Control Program (TUNMES-EAECP) in 2013. The TUNMES-EAECP aimed to determine the incidence of mesothelioma in Turkey, to identify asbestos exposed villages and MM incidence in the rural side, and to create a risk map for environmental asbestos exposure in Turkey.

## 2. Methods

### 2.1. Organization

The TUNMES and EAECP were prepared and performed by TMWG and the Turkish Public Health Institute under the coordination of the Eskisehir Osmangazi University Lung and Pleural Cancers Research and Clinical Centre. The TMWG consisted of 41 researchers; 34 faculty members, two occupational medicine specialists, two mineralogy professors, and three administrative researchers from the Turkish Ministry of Health.

### 2.2. Detection of Mesothelioma Cases

To initiate the TUNMES study organization, 30 provinces that were known to be regions where most of the mesothelioma cases emerged were selected. The mean population of these provinces for five years (2008–2012) was 49,530,708, which constituted 66.8% of the total 81 provinces (74,093,345) in Turkey. MM patients living in the remaining 51 provinces were referred for diagnostic and therapeutic advices to these 30 provinces. Definite histopathological diagnosis and appropriate treatment options for MM can be performed in hospitals in those selected provinces.

In hospitals across these 30 provinces, patients diagnosed with MM (under the code C45.0–C45.9) between 2008 and 2012 (for five years) were identified with approbation. Information on the determined patients was uploaded in a file specifically prepared by the TMWG documenting their names, ages, genders, dates of diagnosis, birth places, and the provinces where they were diagnosed, together with the addresses of their accommodation. Patients with dual entries and missing information were excluded. The cases were checked in the Central Registry System (MERNIS) one by one, according to their identity, name, age, birth place, registration numbers, and addresses, together with their national identity numbers by authorized health staff. Patients with contradictory information were excluded. Consequently, deceased cases were also identified, with their dates of death and ages determined by the automation system of the hospitals using national identity numbers, which were then verified by their National registers.

For this study, the Ministry of Health allowed the examination of patient records (Approval number: 23776858/157.02). The study was conducted in accordance with the Declaration of Helsinki, and the protocol was approved by the Ethics Committee of Eskisehir Osmangazi University (Project identification code PR-12-03-30-01; 30/03/2012).

### 2.3. Determination of the Villages with Asbestos Exposure

A two-step method was followed for identifying asbestos-exposed villages:

(1) In the EAECP studies, a “from case to the field method” was used to determine the villages where there had been asbestos exposure. After obtaining the final records of the cases with MM, the cases of the people born in villages/rural areas, or the places where they used to live were determined, and the villages where people in these cases were born were included to the list of villages from which soil samples needed to be taken.
(2) Health administrators performed a face-to-face survey with the governors of villages where cases were identified using a questionnaire investigating any prior or current exposure of asbestos in that particular village. If any asbestos exposure was reported, that village was also included in the above-mentioned list. Consequently, this list was submitted to both investigators and provincial health administrators of those villages.

### 2.4. Collection of Soil Samples from the Villages

Technical personnel collecting soil samples from these rural areas were trained to “recognize risky soil mixed with asbestos” by clinicians, epidemiologists, and mineralogists in a training program. After training, guidelines were prepared for technical personnel to recognize soil mixed with asbestos, and to collect samples in the rural areas. The information was distributed to the technical personnel as a leaflet, and was uploaded on the website “www.turkiyemezotelyoma.org”. The guide mainly included visual information describing soil mixed with asbestos, its features, and a method of collecting samples from houses, roofing, or mounds.

Trained teams from the provincial directorate of public health visited villages that were considered as at risk of asbestos exposure, and collected soil samples. The sites for sample collection included walls of the houses, roofs, and other areas at risk of asbestos exposure in those villages.

### 2.5. Mineral Analysis of the Soil Samples

The soil samples were delivered to Eskisehir Osmangazi University Lung and Pleural Cancers Research and Clinical Center and coded based on the provinces, districts, villages, areas, and individual houses where samples had been collected. The presence of asbestos in the samples was examined at the Scientific and Technological Research Council of Turkey (TUBITAK), Marmara Research Centre, Material Institute for Mineral Analysis using XRD (X-ray diffractometer), and asbestos was specified based on its sub-types. Analyzed samples and X-ray diffractogram patterns were stored.

### 2.6. Calculation of Incidence Rate of Malignant Mesothelioma

Information regarding the populations of the villages with asbestos exposure were obtained from the official websites www.yerelnet.org.tr and www.nufus.com, and included data from the Turkish Statistical Institute. The incidence was calculated by dividing the number of total cases by the mean population of Turkey in the specified five years.

Cases were stratified by place of birth: rural or urban. Those born in rural areas were further categorized by the absence or presence of asbestos in the village soil. The group made up of the population that had been living or were born in rural areas where soil contained asbestos was considered as the “population living in rural areas, where there is still asbestos exposure”.

Direct Standardized Average Annual Mesothelioma Incidence Rates (AMIRs), and 95% Confidence Interval (CI) adjusted to the World Health Organization (WHO) standard population (WHO 2002, World Health Statistics) were calculated by Poisson’s distribution [13].

### 2.7. Risk Map of Turkey for Environmental Asbestos Exposure

The incidence rate of MM for each province and the estimated number of cases in each province were calculated based on the province where the cases originated. Estimates of the population exposed to standardized incidence ratios were used to compare the cases observed with those expected to develop MM based on the age-specific rates of the Turkish cities or municipalities. Standardized incidence ratios were used to compare the observed cases to those expected based on the age-specific rates of each province. The relative risk (RR) was calculated with the ratio of expected values to the observed values. After the relative risk (RR) calculation, the identification of provinces at risk was determined based on the following criteria: (a) all showing RR > 1; and (b) 95% CI for RR entirely above 1 [14].

### 2.8. Estimates of the Population Exposed to Asbestos in Rural Turkey and the Number of Mesothelioma Cases between 2013 and 2033

To estimate the number of cases expected to develop mesothelioma between 2013 and 2033 among the population living in rural areas where there is still asbestos exposure, the following method was used. In brief, the number of subjects exposed to asbestos was identified based on age, and considering the births, deaths, and migrations in the next 20 years. Based on the data of the Turkish Statistical Institute, rough birth and mortality rates up to 2033 were utilized. Births and deaths in the selected populations were estimated based on the number of births and deaths expected from the annual projected population. The projected volume of migration was obtained based on the projected population for each year, with a decrease of the figure (21.8%) being calculated by the proportion of migration to provinces from villages and to villages from villages in 2000, to the population in 2000. The expected number of MM cases was calculated by multiplying the population as obtained by projection to the incidence of the environmentally exposed group in rural areas with continuing exposure.

### 2.9. Statistical Analysis

All mesothelioma case records were transferred into SPSS 15.0 program (SPSS Inc., Chicago, IL, USA) and Microsoft Excel 7 (Microsoft Corporation, Washington, DC, USA) and analyzed. Data were presented as mean (standard deviation and min-max values) in the quantitative data and as percentiles in qualitative data. The mortality rates were compared with the rate of the selected category by calculating relative risk (RR) and 95% CI.

The groups were compared using the chi-square test. The mean and median survival times were identified according to their diagnosis dates for all of the dead cases. Survival analysis was performed with the Kaplan-Meier method, and were compared with the log-rank test.

## 3. Results

### 3.1. Results of Turkey’s National Mesothelioma Surveillance

According to the TUNMES findings, the number of confirmed MM cases within the 5-year period was 5617. The distribution of these cases by disease characteristics and gender is presented in Table 1. There was no difference in the distribution of cases based on year or gender. The mean ages (± SD) (range) of men, women, and overall cohort was 62.0 ± 13.1 (20–96), 61.3 ± 13.7 (21–95), and 61.7 ± 13.4 years, respectively. The number of patients below the age of 55 and above 75 was 1614 (28.7%) and 1051 (18.7%), respectively. Males and females were not different in terms of annual distribution of MM or age groups.

Considering the number of cases, the men to women ratio in overall Turkey was identified as 1.36 (3241:2376).

Among the 5617 mesothelioma cases, 3738 of the cases had lived in rural areas while 1879 (33.5%) had not. The mean age of the group living in rural areas was detected as 62.3 ± 13.2 years. The number of men and women with rural area history was different. The ratio of men among the cases who did not live in rural areas was significantly high (*p* ˂ 0.05) (Table 1). The men to women ratio was 1.31 for the population living in rural areas, and 1.47 for those not living in rural areas.

During the study period, mortality among men was higher than that of women (*p* < 0.001). The mean age of dead cases was 63.7 ± 12.8 (20–96) years, and the mean age of patients who were alive was 58.4 ± 13.7 (24–95) years (*p* < 0.001). The mean and median survival times (95% CI) of the cases were 11.4 (95% CI 11.0–11.9) and 8 (95% CI: 7.6–8.4) months, respectively.

While most of the cases (96.2%) were pleural MM, peritoneal mesothelioma cases were more likely to be women (*p* < 0.001).

### 3.2. Results of Environmental Asbestos Exposure Control Program

The number of villages whose soil samples required analysis for asbestos risk was 1236 across 58 provinces. However, sampling was not performed in 218 of these villages as provincial governors detected neither the use of asbestos-contaminated soil, nor any building plastered with asbestos-containing material. A total of 1251 soil samples out of 1018 villages were collected from the internal and external walls of houses, risky roofing, and soil mounds.

The XRD analysis of the 1251 samples resulted in 514 soil samples that were determined to have chrysotile (70%), tremolite (20%), or mixed (10%) asbestos fibers.

**Figure 1:**
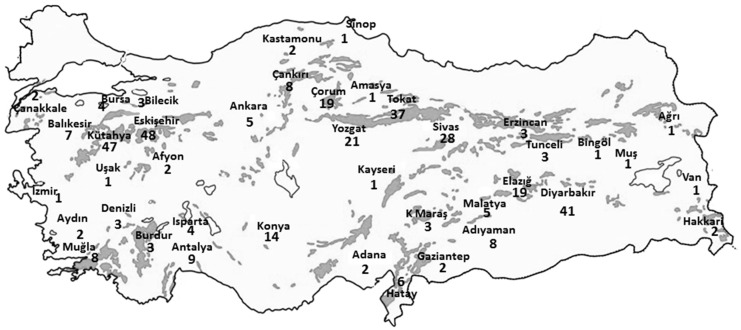
Map of Turkey showing the distribution of villages where asbestos exposure was definite, attached to the provinces where soil samples were taken for asbestos exposure examination based on ophiolites settlement in Turkey. The grey areas represent ophiolites and the figures represent village numbers. This map is reused with the kind permission of Prof. Dr. Aral Okay (Istanbul Technical University, Turkey).

When the registries were checked, it was observed that these 514 soil samples were taken from 379 villages belonging to 41 provinces. Therefore, we decided that asbestos exposure was still ongoing in a total of 379 villages (Figure 1). The distribution of the villages where asbestos exposure continues was determined from the birth or current living places of the cases with MM, are concordant with the presence of ophiolites in soils in Turkey.

### 3.3. The Incidence Rate of Malignant Mesothelioma

**Figure 2:**
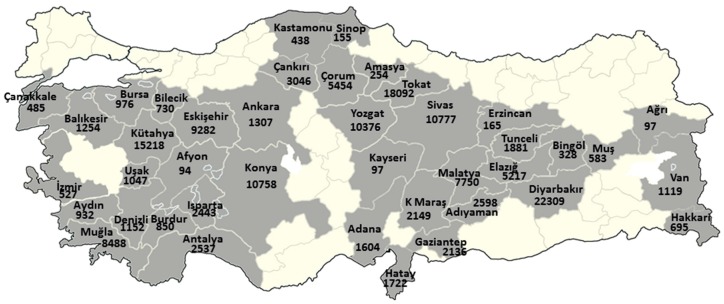
Population living in rural areas, where there is still asbestos exposure ongoing.

The total number of people living in 379 villages/rural areas where asbestos exposure was detected was 158,068. The distribution of the villagers by provinces is provided in Figure 2.

The number of villages where the number of MM cases was higher than expected, but did not have soil sampling as the provincial governors reported no asbestos-contaminated soil and all of the houses had modern plasters, paints, and roof coatings, was 174. The number of inhabitants in these villages was 69,000.

The crude and standardized mesothelioma rates for Turkey determined in the TUNMES study are presented at Table 2.

**Figure 3:**
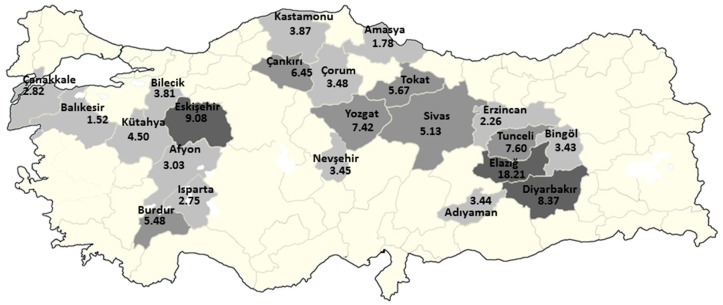
Risk mapping of asbestos exposure-induced mesothelioma in the rural areas of Turkey. Numbers represent Risk Ratio.

In total, 5617 cases were from 58 provinces; however, 98.3% of these cases belonged to the 30 provinces where TUNMES was organized. The provinces were divided into five categories that were based on the RR of mesothelioma (Table 3). The provinces based on the RR of mesothelioma are shown in Figure 3.

The risk of MM was lower in women than in men, and increased gradually in both men and women at ages 40 and over when compared to younger inhabitants. The risk of MM was 11 times higher in the group where asbestos was detected in their village than in the other group. The risk of MM was higher in the provinces where TUNMES was organized than other regions.

### 3.4. Estimates of the Population Exposed to Asbestos in Rural Areas of Turkey, and the Number of Mesothelioma Cases between 2013 and 2033

Out of a population composed of 158,068 people who continue to be exposed to asbestos in 379 villages, the number of mesothelioma cases expected between 2013 and 2033 was estimated as 2511.

## 4. Discussion

In this study, we tried to determine the incidence of MM in Turkey, the frequency of environmental asbestos exposure, the identification of rural areas where asbestos exposure is still continuing, and a risk map for Turkey. We found that the AMIR was 2.33/100,000 person/year. Asbestos exposure is still ongoing in 379 villages with 158,068 inhabitants. If this exposure is not abolished, we expect at least 2511 new MM cases from 2013 to 2033.

Asbestos exposure in rural areas, and the resulting mesothelioma and other relevant diseases, have been addressed in terms of their risk for the concerned region in the regional studies conducted up to now; and accordingly, these constitute important health problems in some regions of Turkey [3,4,8,9,15]. With regard to occupational asbestos exposure, there is almost no information for Turkey overall.

In the TUNMES data, a total of 5617 MM cases (3214 men and 2376 women) were detected in Turkey between 2008 and 2013 (Table 1). The distribution of cases by exposure years or age groups did not show any difference between genders.

The mean age of the patients at diagnosis was 62.3 years among those exposed to asbestos in rural areas. The age at diagnosis in cases of environmental asbestos exposure was lower than those from occupational exposure in the published series [3,4,15,16,17], which may be explained by the introduction of exposure as early as birth. In a study from the central part of Anatolia with 95.8% of the patients with environmental asbestos exposure, a large proportion of the patients (55.6%) were aged under 60 years, with 8% aged 70 years or over. The male to female ratio was 1.06, and the median age was 55.8 years for all of the patients [18]. In the United States, the median age at diagnosis is 74 years, as most MMs develop from occupational exposure [6,7,19]. From 1999–2005, MMs in individuals younger than 55 years represented only 6.7% [19]. During 1999–2015, a total of 45,221 deaths with malignant mesothelioma, of which 16,914 (37.4%) occurred among persons aged 75–84 years [6]. The authors of the study stated that the elevated rates of MM in younger individuals (younger than 55 years old) in combination with a sex ratio of 1:1 (as mentioned above in this age group) suggested the environmental exposure to mineral fibers [19]. In fact, 28.7% of MM cases in this study were below 55 years old, with 18.7% above 75 years old.

In cases of environmental exposure, as the patient may be exposed to asbestos from birth, the ‘latency period’ can be equivalent to the age of the patient at diagnosis. Therefore, the latency period for our patients with environmental asbestos exposure was regarded as 62.3 years, which was the same as the age of diagnosis. The latency period in the occupational exposure series has been generally reported as 30–40 years [20,21]. However, the instantaneous exposure dose from environmental exposure in rural areas was relatively lower than that in the occupational exposure series, albeit a similar cumulative dose [22,23]. In occupational asbestos exposure, it begins at around the age of 25, where the individual is exposed to highly escalating doses in one-third of the day during working hours, and remains exposure-free for the rest of the day [23]. This difference may cause a longer latency period with an onset of disease at a younger age.

A total of 3738 people were born or lived in rural areas/villages where there has been asbestos exposure. The percentage of women living in rural areas was significantly higher than that of men (Table 1), which may be attributed to the potentially higher frequency of occupational asbestos exposure among men. The male to female ratio in this group was 1.31, as compared to 1.47 of those not living in rural areas. This ratio was a distinctive feature of environmental asbestos exposure given that men and women during their daily village life are exposed to asbestos in a similar manner and duration. Therefore, the similarity in this male to female ratio indicates a comparable risk of these two groups in terms of environmental asbestos exposure [16,17,18]. In fact, the decline in the male to female ratio to <3:1 in industrialized countries is regarded as an indicator of environmental exposure to mineral fibers [19].

The annual average number of cases who were born or lived in rural areas/villages was 747 (3758/5); however, this figure was stated as 415 for the year 2000 in the study by the Ministry of Health [24]. This alteration indicated the elevated number of MM cases in Turkey, and may have been due to several factors including a latency period up to 50–60 years in mesothelioma cases living in rural areas [16,17,18], increased access to health care in the past ten years, improvements in the diagnosis and treatment facilities, and the increased advantage of digital records.

The ratio of cases who were dead during the study period was 62.2%; this ratio was in agreeance with the mesothelioma survival time of the cases diagnosed in a consecutive 5 year-period [25]. The mean survival of the cases was determined as 11.4 months, and the median survival was 8 months. This information also complied with information obtained on mesothelioma prognosis [25,26,27].

Regarding the number of mesothelioma cases determined for Turkey overall for the period between 2008 and 2012, the standardized incidence rate of MM was 2.88/100,000 person/year; 1.86/100,000 person/year; and 2.33/100,000 person/year among men, women, and overall in Turkey, respectively (Table 2). These rates can be regarded as critical when considering the annual mesothelioma incidence in other industrialized countries. The number of deaths from MM was reported to be 2597 in 2015 in the USA [6]. About 10,000 MM cases are estimated to exist annually across the population of Western Europe, Scandinavia, North America, Japan, and Australia [28]. While the annual number of mesothelioma cases is estimated at 2361 in the UK, this is expected to peak around 2300–2600 cases between 2017 and 2018 [29,30]. The number of mesothelioma cases per year is around 600 in Australia [31]. However, these rates have been shown to be much higher in cohorts consisting of those in direct asbestos exposure, i.e., employees working in production/processing facilities reached 19–122.4/100,000 person/year [23].

The incidence of MM in villages where asbestos exposure is still present was 79.94, 66.92, and 73.42 per 100,000 person/year among men, women, and overall, respectively (Table 2). These rates are as high as those previously published in Turkey, and also those regarding occupational asbestos exposure in industrialized countries.

The number of villages where asbestos-contaminated soils were used for plastering, painting, or roof coatings was 379, as determined by the EAECP data. It is noteworthy that the villages with a high risk of asbestos exposure showed a suitable settlement distribution near the ophiolites (Figure 1). These villages were distributed across 41 provinces with a total of 158,068 inhabitants, according to the statistics provided by the Turkish Statistical Institute. These villagers, who are continuously exposed to asbestos, are clearly a priority in terms of taking preventative measures (Figure 2).

The risk mapping indicated that Elazig, Eskisehir, and Diyarbakir were the riskiest provinces of Turkey, where the RR was above eight. There were also seven provinces with a RR above 4.5, and 22 provinces with a RR above 1.5. In fact, this was expected when considering previous reports and the distribution of the cases in the study (Figure 3). While there no village in Nevsehir that has recorded asbestos exposure, the presence of erionite in the Cappadocia region also increased the risk for MM in this city. Indeed, this finding is critical for health policy, the necessary development of preventive measures in these cities, and the further strengthening of diagnostic and therapeutic facilities regarding MM.

**Figure 4:**
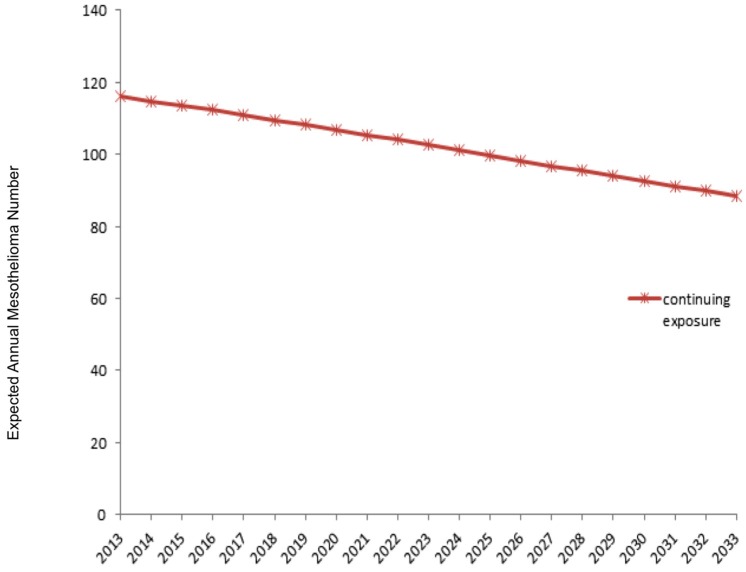
New mesothelioma cases expected among subjects who will continue to be exposed to asbestos after 2013.

The stay period of the registered population in villages where there has been asbestos exposure of whether they live in the village for the whole year or only some months of the year is unknown. Therefore, it is not possible to determine the cumulative asbestos exposure dose of these people individually. However, periodical stays will only affect the volume of risk and does not eliminate the existence of the risk. It is well-known that mesothelioma and the risk of lung cancer is higher when the cumulative dose of asbestos fiber exposure is high, but there is no reliable threshold exposure dose for the emergence of the disease. Furthermore, risk is also present in low doses [18,30]. Accordingly, regardless of the period of stay, living in a village with asbestos exposure is sufficient to be considered as at risk. If the exposure for these 158,068 people is to be consistent between 2013 and 2033, the projected number of new MM cases is likely to be 2511 (Figure 4). Cessation of this exposure in a short period with probable precautions will most likely provide substantial risk reduction.

As mentioned above, the number of expected MM cases did not correspond to all of the MM cases throughout Turkey during that period of time since there a population had been exposed to asbestos in rural areas for a risky period of time, i.e., they had been exposed to asbestos for a duration enough to present a risk for MM, but are now unexposed due to the termination of use of asbestos-contaminated soil in recent years. The estimation of potentially new cases do not appear to be possible without determining the exposure duration of this population. Furthermore, since the prohibition of asbestos use was only established in 2010, there are still occupationally exposed people within the community. Similarly, the incidence of new cases among these is not likely to be predictable.

As identified by the TUNMES data, 1879 of the total cases had not lived in rural areas, and therefore, the work places of these cases might be specified as high risk in terms of occupational asbestos exposure. These workplaces should be identified on the basis of the number of people, and examined in detailed by experts with respect to current or previous asbestos exposure [32]. Certainly, the work places of all the cases would not be determined to have asbestos exposure; however, the identification of the workplaces in these cases would enable the documentation of workplaces with asbestos exposure in Turkey, which will break new ground in terms of efforts towards occupational diseases, and occupational health and safety. It is clear that the results obtained in the plan work form a comprehensive basis to make assessments for the first time for all of Turkey, based on reliable data regarding occupational health. Assessments made with the information from the TUNMES results that also considered the constraints are as follows: Approximately 500,000 tons of asbestos was used in Turkey from 1983 until 2010 when asbestos use was completely banned. Those working with these products (including these amounts) will be exposed to asbestos for at least the next 30–40 years (The Report of Specialization Commission of the State Planning Organization of Turkey, 1996, 2001, 2009–2013). When considering the amount of asbestos used in the industry, it is obvious that exposure during the maintenance, repair, and disassembly of these products will lead to the emergence of relevant diseases in the next twenty years unless efficient measures are taken.

Our study had several limitations. The cases used in the study were derived from hospital records based on diagnostic entries. Patients with ICD45 in Turkey are generally mesothelioma patients diagnosed by immunohistochemical staining; however, in this study, researchers could not confirm the histopathological diagnosis of 5617 cases due to a high number of patients and technical reasons. Additionally, despite a higher incidence of MM than expected, we were not able to identify the presence or absence of asbestos contaminated soil in 179 villages based on statements from regional administrators. Given that there has been high population mobility in Turkey especially in the past three decades, we were only able to identify patients with environmental asbestos exposure, but was not able to collect any data regarding occupational exposure.

## 5. Conclusions

In conclusion, asbestos exposure in rural areas remains a critical problem in Turkey. This study is the first time that the size of the problem and risk of MM was comprehensively identified on a nationwide basis for Turkey. In addition, people at risk were also determined. Our findings allow for both the initiation of preventive measures to avoid environmental asbestos exposure and improvements to early diagnosis and effective therapy by following-up closely with at risk individuals. These cohorts also form the scientific foundation for future MM research in the world where there is continuing asbestos exposure. Although it is not officially described today, it has emerged that the MM related to occupation should now be expected as a problem.

## Figures and Tables

**Table 1:** Distribution of Turkey’s National Mesothelioma Surveillance detected cases by disease characteristics and gender.

Variables	Male (*n* = 3241) *n* (%)	Female (*n* = 2376) *n* (%)	Total (*n* = 5617) *n* (%)	*p*
Years				0.246
2008	570 (17.6)	422 (17.8)	992 (17.7)	
2009	658 (20.3)	455 (19.1)	1113 (19.8)	
2010	606 (18.7)	498 (21.0)	1104 (19.7)	
2011	686 (21.2)	501 (21.1)	1187 (21.1)	
2012	721 (22.2)	500 (21.0)	1221 (21.7)	
Age (years)				0.071
<40	169 (5.3)	154 (6.5)	323 (5.8)	
40–49	385 (11.9)	309 (13.0)	694 (12.4)	
50–59	789 (24.3)	573 (24.1)	1362 (24.2)	
60–69	905 (27.9)	603 (25.4)	1508 (26.8)	
70+	993 (30.6)	737 (31.0)	1730 (30.8)	
Born or living in the rural area				0.04
Yes	2122 (65.5)	1616 (68.0)	3738 (66.5)	
No	1119 (34.5)	760 (31.0)	1879 (33.5)	
Vital status				<0.001
Dead	2097 (64.7)	1398 (58.8)	3495 (62.2)	
Alive	1144 (35.3)	978 (41.2)	2122 (37.8)	
Site of lesion				<0.001
Pleura	3117 (96.2)	2225 (93.6)	5342 (95.1)	
Peritoneum	110 (3.4)	136 (5.7)	246 (4.4)	
Pericardium	14 (0.4)	15 (0.7)	29 (0.5)	

**Table 2:** Crude and standardized mesothelioma incidence rates.

	Crude Incidence Rate Annual per 100,000 (95% CI)	Standardized Incidence Rate * Annual per 100,000 (95% CI)
Turkey		
Male	1.86 (1.79–1.89)	2.88 (2.86–2.89)
Female	1.49 (1.43–1.56)	1.86 (1.85–1.87)
Total	1.84 (1.79–1.92)	2.33 (2.32–2.34)
Villages with continuing asbestos exposure **		
Male	79.94 (67.64–92.17)	87.27 (87.21–87.33)
Female	66.92 (55.84–78.00)	68.44 (68.39–68.49)
Total	73.42 (65.30–81.67)	79.00 (78.94–79.06)

CI = Confidence interval; * = Standardized according to WHO-2000 population; ** = Population living in rural areas, where there is still continuing asbestos exposure.

**Table 3:** Malignant mesothelioma mortality rate and Risk Ratio (95% CI) by gender, age groups, presence of asbestos in the village, and organization.

Variables	Rate Annual per 100,000	Risk Ratio	95% CI
Gender			
Male	1.86	1	
Female	1.49	0.80	0.76–0.84
Male			
<40	0.27	1	
40–49	1.23	4.56	3.76–5.53
50–59	2.83	10.48	8.77–12.52
60–69	4.55	16.85	14.12–20.11
≥70	6.39	23.67	19.90–28.16
Female			
<40	0.26	1	
40–49	1.36	5.23	4.58–5.97
50–59	3.37	12.96	11.48–14.63
60–69	5.93	22.81	20.23–25.72
≥70	8.70	33.46	29.71–37.68
Asbestos in the village			
No	0.75	1	
Yes	8.08	10.77	9.14–12.69
TUNMES Organization			
No	0.37	1	
Yes	1.40	3.78	3.34–4.28

## References

[1] Harber P., Gee J.B.L. (2015). Clinicians’ approach to mesothelioma. Malignant Mesothelioma.

[2] Schuhmann M., Brims F.J.H., O’Reilly K.M.A. (2011). Asbestos-Related Lung Disease: An Update. Clin. Pulm. Med..

[3] Metintas S., Metintas M., Ucgun I., Onder U. (2002). Malignant mesothelioma due to environmental exposure to asbestos. Chest.

[4] Selçuk Z.T., Cöplü L., Emri S., Kalyoncu A.F., Sahin A.A., Bariş Y.I. (1992). Malignant pleural mesothelioma due to environmental mineral fiber exposure in Turkey. Analysis of 135 cases. Chest.

[5] Metintaş M., Hillerdal G., Metintaş S. (1999). Malignant mesothelioma due to environmental exposure to erionite: Follow-up of a Turkish emigrant cohort. Eur. Respir. J..

[6] Mazurek J.M., Syamlal G., Wood J.M., Hendricks S.A., Weston A. (2017). Malignant mesothelioma mortality—United States, 1999–2015. MMWR Morb. Mortal. Wkly. Rep..

[7] Carbone M., Ly B.H., Dodson R.F., Pagano I., Morris P.T., Doğan U.A., Gazdar A.F., Pass H.I., Yang H. (2012). Malignant mesothelioma: Facts, myths and hypotheses. J. Cell Physiol..

[8] Metintas M. (2015). Turkey asbestos control strategic plan final report. Turkish Thorac. J..

[9] Şenyiğit A., Babayiğit C., Gökırmak M., Topcu F., Asan E., Coskunsel M., Işık R., Ertem M. (2000). Incidence of malignant pleural mesothelioma due to environmental asbestos exposure in the southeast of Turkey. Respiration.

[10] Baris Y.I., Grandjean P. (2006). Prospective study of mesothelioma mortality in Turkish villages with exposure to fibrous zeolite. J. Natl. Cancer Inst..

[11] Metintas M., Hillerdal G., Metintas S., Dumortier P. (2010). Endemic malignant mesothelioma: Exposure to erionite is more important than genetic factors. Arch. Environ. Occup. Health.

[12] Institute for Health Metrics and Evaluation (2015). The Global Burden of Disease: Generating Evidence, Guiding Policy.

[13] Ahmad O.B., Boschi-Pinto C., Lopez A.D., Murray J.L.C., Lozano R., Inoue M. (2001). GPE Age Standardization of Rates: A New Who Standard.

[14] Corfiati M., Scarselli A., Binazzi A., Di Marzio D., Verardo M., Mirabelli D., Gennaro V., Mensi C., Schallemberg G., Merler E. (2015). Epidemiological patterns of asbestos exposure and spatial clusters of incident cases of malignant mesothelioma from the Italian national registry. BMC Cancer.

[15] Bayram M., Dongel I., Bakan N.D., Yalcin H., Cevit R., Dumortier P., Nemery B. (2013). High risk of malignant mesothelioma and pleural plaque in subjects born close to ophiolites. Chest.

[16] Metintas M., Metintas S., Ak G., Erginel S., Alatas F., Kurt E., Ucgun I., Yildirim H. (2008). Epidemiology of pleural mesothelioma in a population with non-occupational asbestos exposure. Respirology.

[17] Şahin U., Özturk O., Songür N., Bircan A., Akkaya A. (2009). Observations environmental asbestos exposure in a high-risk area. Respirology.

[18] Metintaş M., Özdemir N., Hillerdal G., Uçgun I., Metintas S., Baykul C., Elbek O., Mutlu S., Kolsuz M. (1999). Environmental asbestos exposure and malignant pleural mesothelioma. Respir. Med..

[19] Baumann F., Carbone M. (2016). Environmental risk of mesothelioma in the United States: An emerging concern-epidemiological issues. J. Toxicol. Environ. Health B.

[20] Lanphear B.P., Buncher C.R. (1992). Latent period for malignant mesothelioma of occupational origin. J. Occup. Med..

[21] Bang K.M., Mazurek J.M., Storey E., Attfield M.D., Schleiff P.L., Wood J.M., Wassell J.T. (2009). Malignant mesothelioma mortality—United States, 1999–2005. MMWR. Morb. Mortal. Wkly. Rep..

[22] Metintas M., Metintas S., Hillerdal G., Ucgun İ., Erginel S., Alataş F., Yıldırım H. (2005). Non-malignant pleural lesions due to environmental exposure to asbestos. Eur. Respir. J..

[23] Hillerdal G. (1999). Mesothelioma: Cases associated with non-occupational and low dose exposures. Occup. Environ. Med..

[24] Barış Y.İ., Akay H., Emri S. (2007). Türkiye’de Asbest ve Erionite ile İlgili Hastalıklar. Toraks. Dergisi..

[25] Sterman D.H., Albelda S.M. (2005). Advances in the diagnosis, evaluation and management of malignant pleural mesothelioma. Respirology.

[26] Batirel H.F., Metintas M., Caglar H.B., Ak G., Yumuk P.F., Yildizeli B., Yuksel M. (2016). Adoption of pleurectomy and decortication for malignant mesothelioma leads to similar survival as extrapleural pneumonectomy. J. Thorac. Cardiovasc. Surg..

[27] Ak G., Metintas S., Metintas M., Yildirim H., Erginel S., Kurt E., Alatas F., Cadirci O. (2009). Prognostic factors according to the treatment schedule in malignant pleural mesothelioma. J. Thorac. Oncol..

[28] Tossavainen A. (2004). Global use of asbestos and the incidence of mesothelioma. Int. J. Occup. Environ. Health.

[29] Hodgson J.T., McElvenny D.M., Darnton A.J., Price M.J., Peto J. (2005). The expected burden of mesothelioma mortality in Great Britain from 2002 to 2050. Br. J. Cancer.

[30] Health and Safety Executive (2011). Mesothelioma Mortality in Great Britain. The Revised Risk and Two-Stage Clonal Expansion Models.

[31] Australian Mesothelioma Registry (2011). 1st Annual Report Mesothelioma in Australia 2011, Safe Work Australia. http://www.mesothelioma-australia.com.

[32] Marsili D., Terracini B., Santana V.S., Ramos-Bonilla J.P., Pasetto R., Mazzeo A., Loomis D., Comba P., Algranti E. (2016). Prevention of Asbestos-Related Disease in Countries Currently Using Asbestos. Int. J. Environ. Res. Public Health.

